# Synthesis of Silver Nanoparticles and Detection of Glucose via Chemical Reduction with Nanocellulose as Carrier and Stabilizer

**DOI:** 10.3390/ijms232315345

**Published:** 2022-12-05

**Authors:** Zhiguo Zhang, Guihua Yang, Ming He, Letian Qi, Xincai Li, Jiachuan Chen

**Affiliations:** State Key Laboratory of Biobased Material and Green Papermaking, Qilu University of Technology (Shandong Academy of Sciences), Jinan 250353, China

**Keywords:** cellulose nanofibrils, cellulose nanocrystals, synthesis, silver nanoparticles, glucose detection

## Abstract

The application of silver nanoparticles (AgNPs) in antibacterial materials, glucose detection, etc., is of broad interest for researchers around the world. Nanocellulose with many excellent properties can be used as a carrier and stabilizer to assist in the synthesis of AgNPs. In this study, cellulose nanofibrils (CNFs) and cellulose nanocrystals (CNCs) were used to assist in the synthesis of AgNPs under the reduction of glucose and detection of glucose concentration under different conditions. Transmission electron microscopy (TEM) analysis showed that the AgNPs in the nanocellulose-AgNPs (NC-AgNPs) system were roughly spherical and randomly distributed on the nanocellulose. In the whole reaction system, when the concentration of nanocellulose is 0.11 mg/mL, the concentration of silver ammonia solution is 0.6 mM, and the mixing time is 2.5 h, according to the UV-Vis analysis, the absorbance of CNF-AgNPs at 425 nm exhibited a good linear relationship (R^2^ = 0.9945) with the glucose concentration range (5–50 μM), while the absorbance of CNC-AgNPs at 420 nm showed a good linear relationship (R^2^ = 0.9956) with the glucose concentration range (5–35 μM). The synthesis of NC-AgNPs can be further developed into a sensor with higher sensitivity and higher stability for detecting glucose concentration and a material with antibacterial effects.

## 1. Introduction

In recent years, as a renewable cellulose material with a wide range of sources, large specific surface area, high strength, good biodegradability and thermal stability, nanocellulose has attracted much attention from all walks of life [1,2]. Moreover, a large number of active hydroxyl groups on the surface of nanocellulose are very suitable for chemical modification [3], which greatly broadens the application scope of nanocellulose. Due to its many excellent properties, nanocellulose can be used as a green matrix material or carrier material to solve the problems of some traditional sensor substrate materials, such as being non-renewable, difficult to degrade naturally, poor mechanical strength or complicated preparation process.

In general, the molecular chains of nanocellulose without special modification and other methods contain a large number of functional groups such as hydroxyl groups, which will have a large number of negative charges in water, and have the function of adsorbing cations, so nanocellulose can be used as a matrix for supporting silver ions [4]. Furthermore, nanocellulose has been found to act as a stabilizer, dispersant and/or reducing agent during the synthesis of AgNPs [5,6,7]. Nanocellulose has a good stabilizing effect on metal nanoparticles, which can promote the nucleation of nanoparticles and prevent their agglomeration [4]. Wang et al. [7] showed that CNF has the ability to significantly improve the stability and dispersibility of AgNPs in physiological media, and also verified that CNF-AgNPs have a certain degree of antibacterial potential. Pawcenis et al. [8] used three different fractions of TEMPO-oxidized nanocellulose as reducing and capping agent for AgNPs. Three different AgNPs-containing nanocomposites were prepared by heat-treating each fraction of nanocellulose in its aqueous suspension with silver ammonia solution. The results show that the three fractions of TEMPO-oxidized nanocellulose, characterized by different water solubility, degree of oxidation and viscosity, can synthesize stable AgNPs in a mild, “greener” manner. Cieśla et al. [9] studied the preparation of CNF from carrot residue as stabilizer and sodium borohydride as reducing agent to synthesize AgNPs in the presence of different silver nitrate concentrations, temperatures and mechanical stirring conditions. The shape and size distribution of AgNPs synthesized by reaction were characterized by different analytical methods. Studies have shown that AgNPs with a size of less than 100 nm can be efficiently synthesized by this reaction, and the nanocellulose obtained from carrot pomace was an excellent stabilizing agent. Liu et al. [10] further modified the CNC prepared by sulfuric acid hydrolysis to prepare carboxylated CNC, used NaBH_4_ to reduce metal cations, and used the carboxylated CNC as the scaffold to synthesize AgNPs. The results showed that the carboxyl and hydroxyl groups of carboxylated CNCs provided coordination to adsorb metal cations and AgNPs, thereby preventing the aggregation of nanoparticles. Ag particles smaller than 10 nm in size were easily prepared and well dispersed. The average size of AgNPs increases with the amount of silver. The Ag/carboxylated CNCs nanocomposites were further used as labels for electrical detection of DNA hybridization.

Glucose (C_6_H_12_O_6_) is an important energy-supplying substance in life, but excessive glucose concentration in the body can lead to many serious and even life-threatening diseases, such as diabetes [11]. Therefore, researchers have been looking for fast, accurate and stable methods to analyze glucose levels in vivo and in vitro [12]. Glucose sensors with high sensitivity, high stability and high accuracy developed by optical [13], electrochemical [14,15] and other methods are of great significance in blood glucose monitoring of diabetic patients. Esmaeili et al. [16] combined polypyrrole (PPy)/CNC composites with glucose oxidase (GOx) to prepare a biosensor for glucose detection. The pyrrole monomer can be polymerized on the high surface area CNC, thereby enhancing the electrocatalytic activity of the composite towards biomolecules. Moreover, the porous structure of PPy/CNC composites is favorable for the adsorption and immobilization of glucose oxidase (GOx) in the pores. This type of sensor has high sensitivity (about 0.73 μA·mM^−1^) and a large dynamic response in the range of glucose concentrations from 1.0 to 20 mM. The improved glucose biosensor has a low limit of detection (LOD) of (50 ± 10) µM, and also excludes interfering substances such as ascorbic acid. Therefore, this type of sensor has a wide range of applications in medicine and other fields. Glucose is a nontoxic and inexpensive food-grade compound, it can also be used as a “green” reducing agent for the exploration and preparation of AgNPs. Therefore, the preparation of AgNPs can be carried out with the aid of nanocellulose using glucose as a reducing agent. Wang et al. [17] used CNC as the substrate and glucose as the reducing agent to synthesize AgNPs at room temperature, thereby preparing a method for detecting glucose concentration changes and antibacterial effects. This detection method can be used to develop visual, quantitative, and higher-sensitivity glucose detection methods, and the CNC-assisted generation of AgNPs have great potential in clinical diagnosis, environmental monitoring and antibacterial applications.

In this study, eucalyptus fiber was used as raw material, CNF was prepared by mechanical grinding and high-pressure homogenization treatment, and CNC was prepared by sulfuric acid hydrolysis. CNF and CNC were mainly used as carriers and stabilizers, and silver ammonia solution was reduced with glucose to explore the preparation of AgNPs under different synthesis conditions. In addition, the size, morphology and color of CNF-AgNPs and CNC-AgNPs under certain conditions, as well as the detection of glucose concentration, were simply compared. In this paper, TEM, X-ray diffraction (XRD), X-ray photoelectron spectroscopy (XPS), thermogravimetric analysis (TGA), colorimetry, UV-Vis spectrophotometer and other methods were used to detect and analyze the AgNPs.

## 2. Results and Discussion

### 2.1. Particle Size, Infrared Spectra and AFM Analysis of Nanocellulose

**Figure 1:**
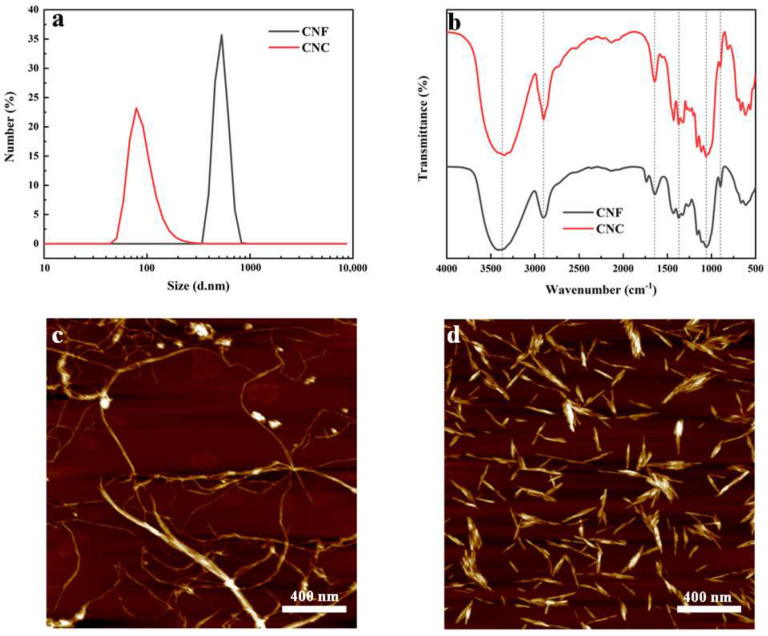
(**a**) Particle size distribution of CNF and CNC, (**b**) infrared spectra of CNF and CNC after freeze-drying, (**c**) AFM image of CNF and (**d**) AFM image of CNC.

The particle size distributions of CNF and CNC are shown in Figure 1a. The Malvern Mastersizer assumes spherical particles when calculating particle size, which means that the particle size distribution should be considered relative since nanocellulose, especially CNF, has a high aspect ratio and is quite different from spherical geometries [18]. The average particle sizes of CNF and CNC prepared in this study are about 529.43 nm and 92.61 nm, respectively. The average zeta potentials of CNF suspension and CNC suspension were about −23.93 mV and −28.53 mV, respectively, indicating good stability.

The infrared spectra of CNF and CNC after freeze-drying are shown in Figure 1b. According to the wavelength values corresponding to the peaks of some functional groups in the infrared spectrum, the peak near 3378 cm^−1^ in the spectrum corresponds to the characteristic peak of stretching vibration of –OH in the cellulose molecule, and the peaks near 2902 cm^−1^ and 1644 cm^−1^ correspond to the stretching vibration peak of C–H and the bending vibration peak of H–O–H absorbing water, respectively, the peaks around 1370 cm^−1^, 1060 cm^−1^, and 898 cm^−1^ correspond to the bending vibration peak of –CH, the stretching vibration peak of C–O, and the vibration absorption peak of anomeric carbon (C1) on cellulose, respectively [19,20,21].

The AFM images of CNF and CNC are shown in Figure 1c,d, respectively. It can be seen from the Figure 1c that CNFs are slender and long fibrils with a certain staggered network structure. The diameter is about 15–25 nm, and the length is hundreds of nanometers to several micrometers. On the one hand, it may be due to the mechanical shear force of the grinding disc of the supermasscollider, which promoted the breakage of the cellulose chains and the nanolization of fibers [22]. On the other hand, in the process of high-pressure homogenization, along with a certain homogenization pressure and homogenization times, the fibers were subjected to shearing force, impact and cavitation generated during the process, and the length and width of the fibers were affected. All of them were significantly reduced, and the fibers were obviously split into microfibrils [23]. Figure 1d shows that the prepared CNCs are generally a short rod-like structure, and the other part has an irregular shape. The short rod-shaped sample is about 10–30 nm in diameter, and the length is mainly distributed in 50–250 nm. The reason for the short rod-shaped sample may be that under the condition of complete hydrolysis, the contact between sulfuric acid and the fiber was relatively sufficient, and the hydrolysis rate of the amorphous region was faster. This situation has led to a reduction in the size of the CNC [24].

### 2.2. TEM Analysis

**Figure 2:**
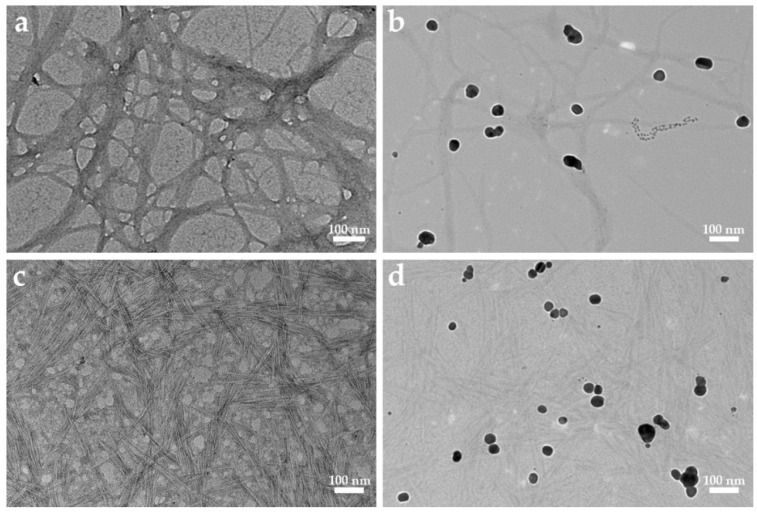
TEM images of the samples. (**a**) CNF, (**b**) CNF-AgNPs, (**c**) CNC, (**d**) CNC-AgNPs.

The TEM images of nanocellulose and NC-AgNPs are shown in Figure 2. As shown in Figure 2a, CNFs are thin and long fibrils with a network structure. Figure 2c shows that the CNCs generally present a short rod-like structure with relatively small diameter and length. Figure 2b,d show that the nearly spherical AgNPs are relatively uniformly dispersed on the nanocellulose surface, and due to the loading and dispersion functions of the nanocellulose, the AgNPs do not show obvious aggregation. Nearly spherical nanoparticles with relatively small average diameters are obtained. However, there are also some nanoparticles with larger diameters and nanoparticles with shapes close to triangles and hexagons (Figure S1). Figure 2b shows the average diameter of AgNPs loaded on CNF is 31.21 ± 7.10 nm. Figure 2d shows the average diameter of AgNPs loaded on CNC is 29.59 ± 9.34 nm.

### 2.3. XRD Analysis

**Figure 3:**
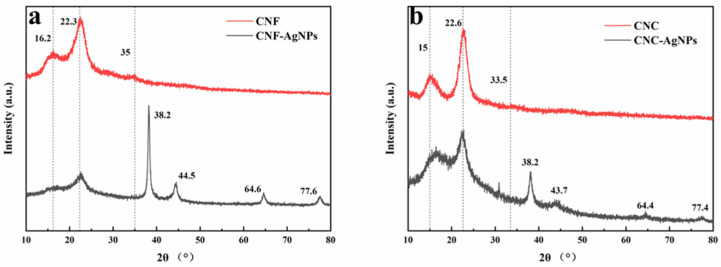
XRD pattern of nanocellulose and NC-AgNPs. (**a**) CNF and CNF-AgNPs, (**b**) CNC and CNC-AgNPs.

The X-ray diffraction patterns of nanocellulose and NC-AgNPs are shown in Figure 3. The diffraction peaks of CNF appeared at 2θ = 16.2°, 22.3° and 35.0°, and the diffraction peaks of CNC appeared at 2θ = 15.0°, 22.6° and 33.5°. They correspond to the (110), (002) and (040) crystal planes of cellulose, respectively, which are typical structures of cellulose I. The relative crystallinity of CNF is about 48.39%, and the relative crystallinity of CNC is about 70.48%. Due to the high shear force generated during the mechanical treatment of the supermasscollider and the high-pressure microjet homogenization treatment, the hydrogen bonding force between the fibrils may be weakened, and the intermolecular hydrogen bonds of cellulose were broken, resulting in the collapse of the cellulose crystal structure, so the crystallinity of CNF was relatively low [25,26]; in the process of preparing CNC by acid hydrolysis, hydrated hydrogen ions penetrated the more accessible amorphous zone of cellulose during hydrolysis and allow hydrolytic cleavage of glycosidic bonds [27]. The crystalline area is relatively stable, and the part destroyed by the acid solution is relatively small, so the proportion of the crystalline area increases, and the crystallinity increases.

According to the XRD patterns of CNF-AgNPs and CNC-AgNPs, they both have characteristic diffraction peaks of nanocellulose, CNF-AgNPs have new diffraction peaks at 38.2°, 44.5°, 64.6° and 77.6°, and the new diffraction peaks of CNC-AgNPs are found at 38.2°, 43.7°, 64.4° and 77.4°. The four new diffraction peaks of the two composites corresponded to the Ag (111), Ag (200), Ag (220) and Ag (311) crystal planes, respectively, indicating that AgNPs are indeed generated in the system.

### 2.4. XPS Analysis

**Figure 4:**
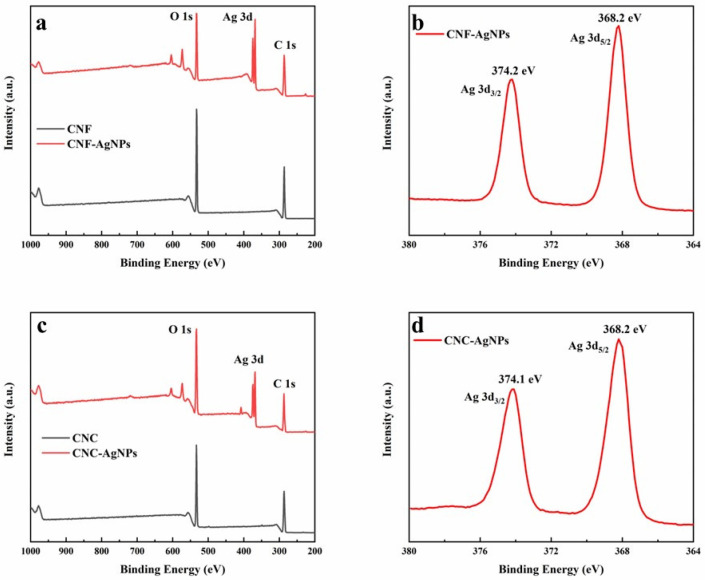
XPS spectra of nanocellulose and NC-AgNPs. (**a**) XPS spectra of CNF and CNF-AgNPs, (**b**) XPS spectrum of Ag 3d of CNF-AgNPs, (**c**) XPS spectra of CNC and CNC-AgNPs, (**d**) XPS spectrum of Ag 3d of CNC-AgNPs.

Figure 4a,c show that the absence of Ag elements in XPS spectra of CNF and CNC appeared in XPS spectra of NC-AgNPs, indicating that AgNPs were successfully synthesized during the reaction. Figure 4b shows that in the XPS spectrum of the Ag region of the CNF-AgNPs, two peaks appeared at 368.2 and 374.2 eV, corresponding to the binding energies of Ag 3d_5/2_ and Ag 3d_3/2_, respectively. Figure 4d shows in the XPS spectrum of the Ag region of the CNC-AgNPs, two peaks appeared at 368.2 and 374.1 eV, corresponding to the Ag 3d_5/2_ and Ag 3d_3/2_ binding energies, respectively [28,29].

### 2.5. Thermal Stability

**Figure 5:**
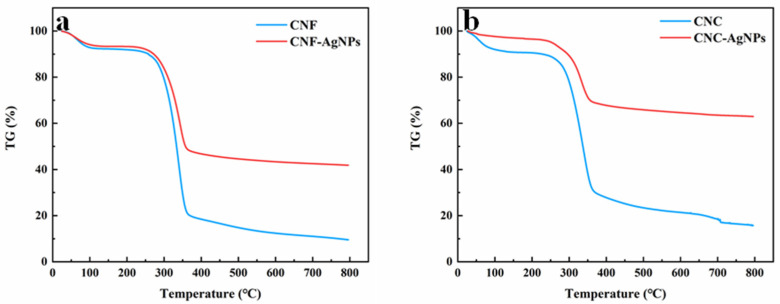
TG graphs of the nanocellulose and NC-AgNPs. (**a**) TG graphs of the CNF and CNF-AgNPs, (**b**) TG graphs of the CNC and CNC-AgNPs.

Thermogravimetric analyses of nanocellulose and NC-AgNPs were performed under nitrogen atmosphere to evaluate their degradation profiles and thermal stability. Figure 5 shows that each sample has two main weight loss stages, below 100 °C and around 300–400 °C, respectively, where the first weight loss stage corresponds to the evaporation of adsorbed water in the sample. For nanocellulose, the second weight loss stage corresponds to decomposition and carbonization of cellulose, and for NC-AgNPs, the second weight loss stage corresponds to the decomposition and carbonization of cellulose, as well as the oxidation of AgNPs. Figure 5a shows that the total weight loss of CNF is about 90.5%, and the TG curve of CNF-AgNPs shows that the total weight loss is about 58.2%. Figure 5b shows that the total weight loss of CNC is about 84.3%, and the TG curve of CNC-AgNPs shows that the total weight loss is about 37.0%. At the same time, the figure also shows that NC-AgNPs have high thermal stability.

### 2.6. Colorimetric and UV-Vis Spectroscopic Analysis

**Figure 6:**
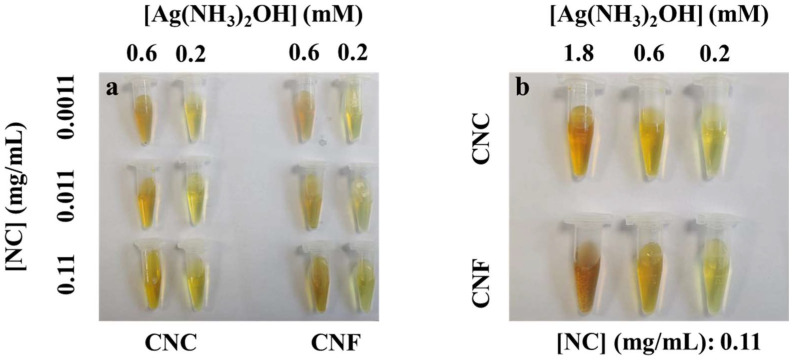
Pictures of NC-AgNPs system after 2.5 h reaction when the glucose concentration is 45 μM. ((**a**), When the concentrations of CNF and CNC in the mixed system are 0.0011 mg/mL, 0.011 mg/mL, 0.11 mg/mL respectively, and the concentrations of silver ammonia solution are 0.6 mM, 0.2 mM, respectively, the corresponding reaction color pictures; (**b**), When the concentration of CNF and CNC in the mixed system are 0.11 mg/mL and the concentration of silver ammonia solution are 1.8 mM, 0.6 mM and 0.2 mM respectively, the corresponding reaction color pictures).

Figure 6 shows that when the glucose concentration in the reaction system is 45 μM and the reaction is carried out for 2.5 h, fixing the concentration of one component of silver ammonia solution or nanocellulose and increasing the concentration of the other component will make the color of the reaction system darker to a certain extent. It may be that the nanocellulose molecular chain prepared in this study has many functional groups such as hydroxyl groups, which are negatively charged in water and are easily complexed with positively charged ions. Nanocellulose is beneficial to the synthesis of AgNPs to a certain extent due to its good dispersibility and stability, as well as providing abundant carriers for silver-containing ions. However, in the absence of glucose in the mixed system, no color change was observed even after 4 h of reaction (Figure S2). It indicates that the nanocellulose prepared in this experiment did not exhibit a “reducing agent” effect under the present experimental conditions.

**Figure 7:**
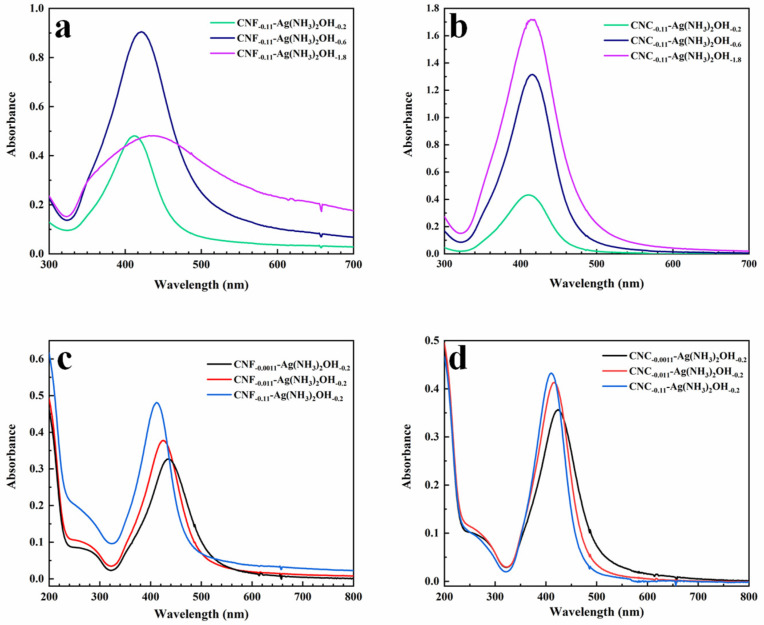
UV-Vis spectra of NC-AgNPs after 4 h reaction. UV-Vis spectra of AgNPs mixed system ((**a**), CNF-AgNPs; (**b**), CNC-AgNPs) obtained at different concentrations of silver ammonia solution (the concentration of nanocellulose is 0.11 mg/mL, the concentration of glucose is 45 μM); UV-Vis spectra of AgNPs mixed system ((**c**), CNF-AgNPs; (**d**), CNC-AgNPs) obtained at different nanocellulose concentrations (the concentration of silver ammonia solution is 0.2 M, the concentration of glucose is 45 μM).

Figure 7 shows the recorded UV-Vis absorption spectra of CNF-AgNPs and CNC-AgNPs after 4 h of synthesis reaction, the dispersion CNF-AgNPs was diluted (1:3.5) with ultrapure water, the dispersion CNC-AgNPs was diluted (1:3.35) with ultrapure water. From the absorption spectrum, a strong absorption peak appears between about 300 and 600 nm with a maximum around 400–450 nm, which is the surface plasmon resonance (SPR) band of AgNPs [30]. This peak and its position indicate that AgNPs were successfully synthesized in this system [31].

It can be seen from Figure 7a,b that when the concentration of nanocellulose (0.11 mg/mL) and the concentration of glucose (45 μM) in the mixed system remain unchanged, the SPR absorption peak shows a certain degree of red-shift with the increase in concentration of silver ammonia solution (0.2 mM, 0.6 mM, 1.8 mM). This may be due to the increased size and number of synthesized AgNPs [32,33]. Additionally, note that in the CNC-AgNPs hybrid system (Figure 7b), the absorption intensity of the SPR band shows an increasing trend, which is consistent with the color change in the system, which indicates an increase in the number of AgNPs supported on nanocellulose. In the CNF-AgNPs system (Figure 7a), when the concentrations of silver ammonia solution are 18 mM and 6 mM, the maximum absorbance value of the former is not greater than that of the latter, which may be due to more agglomeration and sedimentation of CNF and AgNPs during the reaction process, resulting in a certain decrease in the intensity of the SPR peak.

Figure 7c,d show that in the mixed system, in the presence of nanocellulose (0.0011 mg/mL, 0.011 mg/mL and 0.11 mg/mL), when the concentration of silver ammonia solution (0.2 mM) and the concentration of glucose (45 μM) remain unchanged, with the increase in nanocellulose concentration, the SPR absorption peak appears blue-shifted, but its intensity increases. When the system does not contain nanocellulose and other conditions are the same, this trend is still followed (Figure S3). It indicates that the particle size of the generated AgNPs gradually decreases but the number increases, which may be related to steric hindrance, and it shows that nanocellulose may also control the nucleation of AgNPs in the presence of glucose.

### 2.7. Glucose Concentration Detection

**Figure 8:**
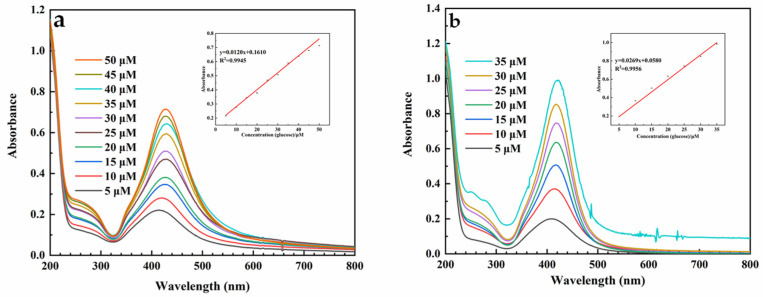
The UV-Vis spectra of the AgNPs mixed system ((**a**), CNF-AgNPs; (**b**), CNC-AgNPs) obtained at different concentrations of glucose and the relationship between the maximum absorbance and the glucose concentration (the concentration of nanocellulose is 0.11 mg/mL, and the concentration of silver ammonia solution is 0.6 mM).

Figure 8 shows that in the mixed system, when the concentration of nanocellulose was 0.11 mg/mL and the concentration of silver ammonia solution was 0.6 mM, the prepared mixed system containing NC-AgNPs was diluted with ultrapure water after 2.5 h of reaction. According to UV-Vis spectra, the maximum absorbance of the mixed system increases with the increase in glucose concentration within a certain range. In the CNF-AgNPs system (Figure 8a), there is a good linear relationship (R^2^ = 0.9945) between the absorbance at 425 nm of the reaction system and the glucose concentration (5–50 μM). In the CNC-AgNPs system (Figure 8b), when glucose concentration ranges from 5 to 35 μM, and there is a good linear relationship (R^2^ = 0.9956) between the absorbance at 420 nm and the concentration of glucose.

## 3. Materials and Methods

### 3.1. Materials

Bleached chemical eucalyptus pulp board (with 87.27% cellulose) was obtained from a paper mill in eastern China. Silver nitrate (AgNO_3_, purity ≥ 99.8%) and Potassium hydroxide (KOH, purity ≥ 85.0%) were purchased from Sinopharm Chemical Reagent Co., Ltd. (Shanghai, China). Ammonium hydroxide solution (AR, 25%~28%) and D-(+)-glucose (ultra pure, purity ≥ 99.5%) were purchased from Shanghai Macklin Biochemical Technology Co., Ltd. (Shanghai, China). Deionized water (made in laboratory). Ultrapure water (made in laboratory, resistivity > 18 MΩ·cm).

### 3.2. Preparation of Nanocellulose

The preparation of CNF was adapted from procedures reported in previously published literatures [34,35,36]. The eucalyptus pulp soaked in deionized water and defibrated was finally diluted to a concentration of 1 wt% and subjected to a certain degree of 10 passes of mechanical grinding with a grinder (Supermasscolloider MK-CA6-5J, Masuko sangyo Co., Ltd., Kawaguchi, Japan). CNF suspension was finally obtained with an M-110EH-30 microfluidizer (Microfluidics, Newton, MA, USA) by passing through 200 μm chamber and 87 μm chamber at a pressure about 1100 bar 10 times.

The preparation of CNC was adapted from procedures reported in previously published literatures [27,37,38]. 180 mL of sulfuric acid (H_2_SO_4_, 64 wt%) was slowly added to 10 g of pulverized dried eucalyptus pulp, stirred appropriately for 75 min in a constant temperature water bath at 45 °C, and then diluted with a large amount of deionized water to terminate the reaction, followed by standing overnight, then the supernatant was removed and the remaining substance was centrifuged several times at 10,615× *g* for 10 min each time. The obtained suspension was dialyzed in ultrapure water until the pH value was nearly neutral and the conductivity was constant at the same temperature. Then, ultrasonic treatment was carried out for 15 min in an ice-water bath.

### 3.3. Preparation of AgNPs

The preparation of silver ammonia solution and the synthesis of the AgNPs were adapted from procedures reported in previously published literatures [4,17]. First, a certain volume of silver nitrate solution (0.1 M) was prepared in a beaker, and 1 wt% dilute ammonia water was gradually added to the beaker with proper stirring until the brown precipitate formed during the process just disappeared. Then, 2 mL of the prepared potassium hydroxide solution (0.8 M) was added to the beaker, and ammonia water was added dropwise again until the solution was clear again. Finally, the silver ammonia solution was diluted with ultrapure water to a final concentration of 18 mM and placed at 4 °C in a dark environment for later use.

**Figure 9:**
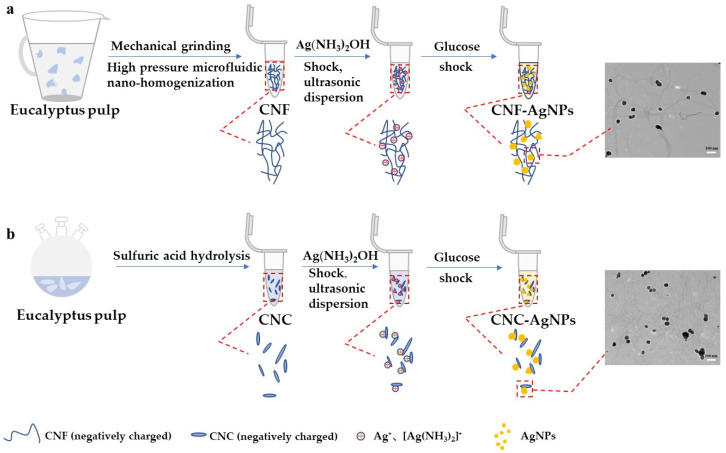
The preparation scheme of AgNPs assisted by nanocellulose as a carrier and stabilizer. ((**a**), CNF was prepared by mechanical method from eucalyptus fiber to assist in the synthesis of AgNPs; (**b**), CNC was prepared from eucalyptus fiber by sulfuric acid hydrolysis method to assist in the synthesis of AgNPs).

Previously prepared suspensions of CNF and CNC were diluted to 0.1375 mg/mL with ultrapure water. The prepared nanocellulose suspension was mixed with a vortex shaker for 10 min, sonicated for 3 min, and the silver ammonia solution was diluted to 6 mM. Finally, a certain volume of nanocellulose suspension was drawn with a pipette and mixed with silver ammonia solution for 3 min, then glucose solution (450 μM) was added, and the mixture was shaken for another minute. The volume ratio of nanocellulose suspension, glucose solution and silver ammonia solution added in this study was 8:1:1. In the preparation of AgNPs in this experiment, the combination of different nanocellulose concentrations and silver ammonia concentrations were recorded as NC_-x_-Ag(NH_3_)_2_OH_-y_, (the x is the concentration of nanocellulose in the mixed system; the y is the concentration of silver ammonia solution in the mixed system). The preparation scheme of AgNPs assisted by nanocellulose as a carrier and stabilizer is shown in Figure 9.

### 3.4. Detection of Glucose Concentration

According to colorimetric method, UV-Vis spectrum and other factors to determine the relatively good concentration of silver ammonia solution and nanocellulose suspension in this experiment, and then added different concentrations of glucose aqueous solution (0.05–1.0 mM) into the mixed system. UV-Vis absorption spectra between 200 and 800 nm were recorded by UV-Vis spectrophotometer to evaluate the sensitivity and good linear range of the glucose determination method.

### 3.5. Characterization Methods

#### 3.5.1. Particle Size Distribution of Nanocellulose

The particle size distribution and zeta potential of nanocellulose suspensions were determined by Zetasizer Nano ZS90 (Malvern Instruments Ltd., Worcestershire, UK). Before the measurement, all samples were diluted to a concentration of 0.015 wt% with ultrapure water, shaken for 10 min, sonicated for 3 min, and measured at 25 °C.

#### 3.5.2. Fourier Transform Infrared Spectroscopy (FT-IR)

The dried samples to be tested were recorded with Fourier-transform infrared (FTIR, Bruker ALPHA, Ettlingen, Germany) to record the spectral information of the samples and conduct infrared spectral analysis of the samples. The measured wavelength range was 4000–400 cm^−1^, the number of scans was 32, and the resolution was 4 cm^−1^.

#### 3.5.3. Atomic Force Microscopy (AFM)

The surface topography and morphology of the nanocellulose samples were observed using an atomic force microscope (AFM, Multimode 8, Bruker, Germany). The samples were prepared into 0.005 wt% CNF suspension and 0.001 wt% CNC suspension, respectively, shaken for 10 min, then sonicated for 3 min, and 1–3 drops of nanocellulose suspension were dropped on a clean mica sheet surface, the samples were dried and used for testing. The AFM images of nanocellulose were finally analyzed and processed by NanoScope Analysis software.

#### 3.5.4. Transmission Electron Microscopy (TEM)

Nanocellulose and NC-AgNPs were tested at the voltage of 200 kV using a JEM-2100 transmission electron microscope (JEOL Ltd., Tokyo, Japan). For TEM detection of nanocellulose, about 7 μL of the dispersed nanocellulose suspension diluted to a concentration of 0.001 wt% was placed on a common carbon support film and dried at room temperature, and then about 1.25 wt% phosphotungstic acid was dropped on the sample for 3–5 min. Dry again at room temperature. For the TEM detection of NC-AgNPs, about 7 μL of a certain concentration of NC-AgNPs mixture were pipetted onto an ultra-thin carbon film and air-dried at room temperature for detection. The dimensions of the nanocellulose and AgNPs were measured from the TEM images using the ImageJ software.

#### 3.5.5. Colorimetric and UV-Vis Spectrophotometer

The color change of the reaction system was observed with the naked eye as the reaction time increased. UV–Vis absorption spectra of the formed AgNPs were recorded on a UV–Vis spectrophotometer (Agilent Cary 8454, Agilent Technologies, Santa Clara, CA, USA) with a scan range of 200–800 nm. Use quartz cuvettes with a 10 mm pathlength. The absorption spectrum of this system was measured after two and a half hours of reaction.

#### 3.5.6. X-ray Diffraction (XRD)

The crystallinity of the samples was tested by an X-ray diffractometer (XRD, D8-ADVANCE, Bruker, Germany) with a working voltage of 40 kV and a working current of 20 mA. XRD data were collected within a 2θ range of 5°–80° at a scanning speed of 20°/min. The crystallinity (%) of each sample was calculated from its X-ray diffraction pattern by Segal’s method [39].

#### 3.5.7. X-ray Photoelectron Spectroscopy (XPS)

The elemental composition and valence distribution information on the surface of the samples were determined using ESCALabXi+ (Thermo Fisher Scientific, Waltham, MA, USA) photoelectron spectroscopy (XPS) instrument. The correction position of C 1 peak was 284.8 eV.

#### 3.5.8. Thermogravimetric Analysis (TGA)

The thermal properties of the samples were investigated by a thermogravimetric analyzer (TGA Q50, TA Instruments, Milford, MA, USA). Samples of about 5–10 mg were heated from around 25 °C to 800 °C at a heating rate of 10 °C/min under nitrogen atmosphere.

## 4. Conclusions

In this study, AgNPs were synthesized by CNF and CNC prepared from eucalyptus pulp fiber as the main carrier and stabilizer, and glucose as the reducing agent. The morphology and structure of NC-AgNPs were characterized by TEM, XRD, XPS, colorimetry and UV-Vis spectroscopy. The TEM analysis showed that the synthesized NC-AgNPs were almost spherical and randomly distributed on the nanocellulose, and the diameters of AgNPs loaded on CNF and CNC are about 25–40 nm and 10–40 nm, respectively. Under the synthesis conditions of 0.11 mg/mL nanocellulose, 0.6 mM silver ammonia and 2.5 h reaction, UV-Vis spectrum showed that the absorbance of CNF-AgNPs at 425 nm had a good linear relationship (R^2^ = 0.9945) with the glucose concentration (5–50 μM). The absorbance of CNC-AgNPs at 420 nm shows a good linear relationship (R^2^ = 0.9956) with glucose concentration (5–35 μM). Nanocellulose used as a carrier and stabilizer is an environmentally, biodegradable and renewable polymer. Glucose as a reducing agent is safe and non-toxic. However, the sensitivity and stability of detecting glucose concentrations need to be improved.

## Data Availability

The data presented in this study are available on request from the corresponding author.

## Supplementary Materials

The following supporting information can be downloaded at: https://www.mdpi.com/article/10.3390/ijms232315345/s1.
