# Supplementary material for: Synthesis of Silver Nanoparticles and Detection of Glucose via Chemical Reduction with Nanocellulose as Carrier and Stabilizer
Source: Int J Mol Sci. 2022 Dec 5;23(23):15345. doi: 10.3390/ijms232315345 (PMC9738211; doi:10.3390/ijms232315345)
Supplement: Supplementary file 1 [file ijms-23-15345-s001.zip › ijms-2004299-supplementary.pdf]

International Journal of
Molecular Sciences

Article

Synthesis of Silver Nanoparticles and Detection of Glucose via Chemical Reduction with Nanocellulose as Carrier and Stabilizer

Zhiguo Zhang, Guihua Yang *, Ming He *, Letian Qi, Xincai Li and Jiachuan Chen

State Key Laboratory of Biobased Material and Green Papermaking, Qilu University of Technology (Shandong Academy of Sciences), Jinan 250353, China
* Correspondence: ygh@qlu.edu.cn (G.Y.); heming8916@qlu.edu.cn (M.H.); Tel.: +86 53189631681 (G.Y.)

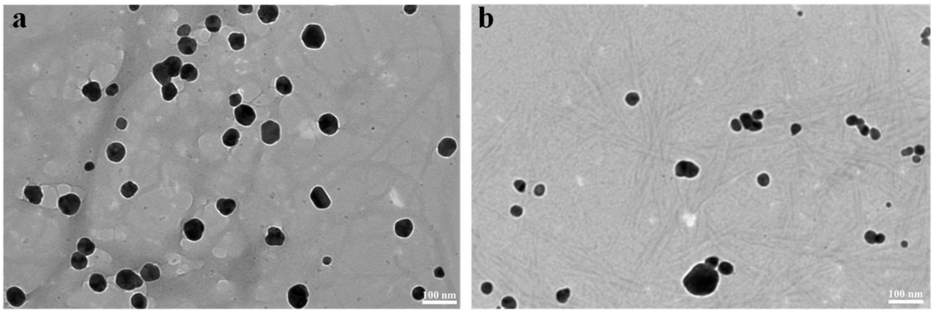

Figure S1. TEM images of the samples. (a) CNF-AgNPs, (b) CNC-AgNPs. In the mixed system, most of the observed AgNPs are spherical or nearly spherical, but there are also some nanoparticles with larger diameters, as well as those with shapes approaching triangles and hexagons.

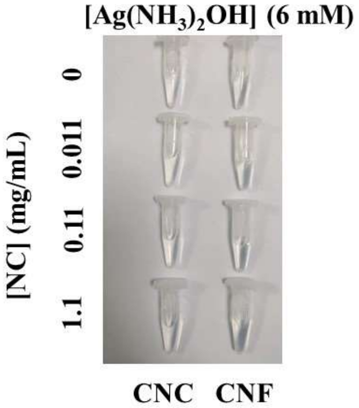

Figure S2. In the mixed system, when the glucose is not added, no color change was observed in the mixed system after 4 h reaction.

International Journal of
Molecular Sciences

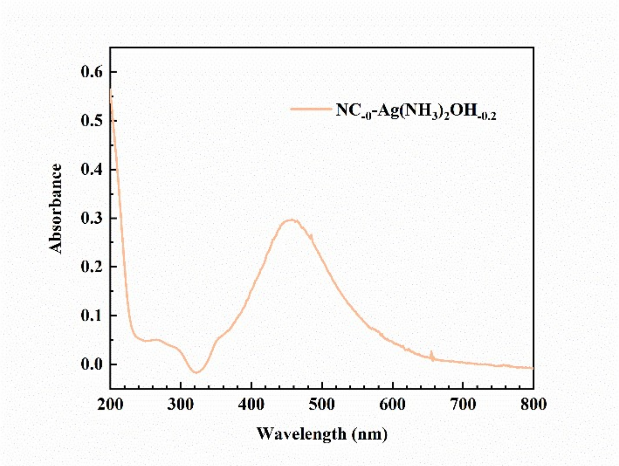

Figure S3. In the mixed system, when the concentration of silver ammonia solution is 0.2 mM and the concentration of glucose is 45 μM, and the nanocellulose is not added, the UV−Vis spectra of the mixed system were detected by dilution (1:3.5) with ultrapure water after 2.5 h reaction.
